# Activity of trabectedin and the PARP inhibitor rucaparib in soft-tissue sarcomas

**DOI:** 10.1186/s13045-017-0451-x

**Published:** 2017-04-11

**Authors:** Audrey Laroche, Vanessa Chaire, François Le Loarer, Marie-Paule Algéo, Christophe Rey, Kevin Tran, Carlo Lucchesi, Antoine Italiano

**Affiliations:** 1 INSERM ACTION U1218, Institut Bergonié, 229 cours de l’Argonne, 33076 Bordeaux cedex, France; 2 Sarcoma Unit, Institut Bergonié, Bordeaux, France; 3 Department of Pathology, Institut Bergonié, Bordeaux, France; 4 University of Bordeaux, Bordeaux, France

**Keywords:** PARP, Trabectedin, Sarcomas, Synergy

## Abstract

**Background:**

Trabectedin has recently been approved in the USA and in Europe for advanced soft-tissue sarcoma patients who have been treated with anthracycline-based chemotherapy without success. The mechanism of action of trabectedin depends on the status of both the nucleotide excision repair (NER) and homologous recombination (HR) DNA repair pathways. Trabectedin results in DNA double-strand breaks. We hypothesized that PARP-1 inhibition is able to perpetuate trabectedin-induced DNA damage.

**Methods:**

We explored the effects of combining a PARP inhibitor (rucaparib) and trabectedin in a large panel of soft-tissue sarcoma (STS) cell lines and in a mouse model of dedifferentiated liposarcoma.

**Results:**

The combination of rucaparib and trabectedin in vitro was synergistic, inhibited cell proliferation, induced apoptosis, and accumulated in the G2/M phase of the cell cycle with higher efficacy than either single agent alone. The combination also resulted in enhanced γH2AX intranuclear accumulation as a result of DNA damage induction. In vivo, the combination of trabectedin and rucaparib significantly enhanced progression-free survival with an increased percentage of tumor necrosis.

**Conclusion:**

The combination of PARP inhibitor and trabectedin is beneficial in pre-clinical models of soft-tissue sarcoma and deserves further exploration in the clinical setting.

## Background

Up to 40% of patients diagnosed with localized soft-tissue sarcoma (STS) will develop metastatic disease [1]. Once metastases are detected, median survival is approximately 12 months, and treatment is mainly based on palliative chemotherapy [2]. Single-agent doxorubicin is the first line standard treatment in this context. Trabectedin (Et-743) has been approved recently in the USA and in Europe for the management of patients with advanced liposarcoma or leiomyosarcoma who have failed to benefit from anthracycline-containing regimen. The 6-month progression-free rate is approximately 35–40% [3–7]. Therefore, the identification of potential agents to combine with this drug to improve patient outcome is crucial.

Even though the exact mechanism of action of trabectedin has not been fully elucidated, previous in vitro studies have demonstrated that trabectedin depends on the status of both nucleotide excision repair (NER) and homologous recombination (HR) DNA repair pathways [8–12]. NER is involved in the repair of DNA lesions induced by ultra-violet light, carcinogens, or platinum-based regimens used in chemotherapy [13]. HR is predominantly involved in the repair of DNA double-strand breaks during the S or G2 phase of the cell cycle using the second undamaged chromosome as a template [14]. Several pre-clinical studies reported that NER-deficient cells were more resistant to trabectedin than their NER-proficient counterparts [8, 9, 11, 12, 15]. Indeed, trabectedin adducts have been suggested to induce a trapping of NER factors, which result in increased levels of cytotoxic DNA damage [12, 13]. ERCC5 (XPG) endonuclease was suggested to be the main NER protein involved in this process [10, 16]. It was also shown that cells deficient in HR are more sensitive to trabectedin than their normal counterparts due to the persistence of DNA lesions and increased formation of replication-dependent double-strand breaks (DSBs) [11]. Interestingly, BRCA1, a key regulator involved in DNA end resection during HR [17], is a marker that is part of a gene signature associated with sensitivity to trabectedin treatment [18]. We have also reported that the status of the *ERCC1*, *ERCC5,* and *BRCA1* genes can predict efficacy of trabectedin in STS patients [19, 20].

PARP-1 recognizes and binds to sites of single-strand DNA breaks (SSBs). In cancer therapeutics, accumulation of SSBs with PARP inhibition leads to the development of DSBs, which require competent HR repair to allow cell survival. PARP has also been shown to be involved in DSB repair pathways. PARP inhibitors (PARPinhs) have been shown to increase the persistence of DNA breaks and cytotoxicity of DNA-damaging agents [21, 22]. Rucaparib is one of the first PARPinhs that have been evaluated in the context of a clinical trial, including clinical trials involving cancer patients [23].

Given that both trabectedin and PARPinh mechanisms of action involve DNA repair machinery, we decided to explore the effects of the combination in soft-tissue sarcomas.

## Methods

### Cells and cell culture

All of the STS cell lines used in this study were derived from human surgical specimens of STS in the laboratory of Pr. Jean-Michel Coindre and Dr Frédéric Chibon (Institut Bergonié, Bordeaux, France) and after obtaining written informed patient consent (Table 1) and Institut Bergonié IRB approval. Each cell line was characterized by array comparative genomic hybridization for every ten replicates to verify that its genomic profile was still representative of the originating tumor sample. Cells were grown in RPMI medium 1640 (Sigma Life Technologies, Saint Louis, MO) in the presence of 10% fetal calf serum (Dutscher, France) in flasks. Cells were maintained at 37 °C in a humidified atmosphere containing 5% CO_2_.

**Table 1:** Antiproliferative activity of trabectedin and rucaparib in soft-tissue sarcoma cells

Cell line ID	Histology	TP53 status	IC50 Trabectedin (nM)	IC50 rucaparib (μM)	Genomic index	ERCC5 mRNA (relative expression level)	ERCC1 mRNA (relative expression level)	BRCA1 mRNA (relative expression level)
IB114	UPS	WT	0.352	1.488	400		+	+
IB115	DDLPS	WT	0.445	1.104	93		+	−
IB111	DDLPS	WT	0.480	1.364	331	+	+	++++
IB133	LMS	Mut	2.64	58.08	328	+	++	
IB134	LMS	Mut	1.92	40.88	340		++	+
IB136	LMS	Mut	1.1	31.35	520	−	+	+
IB112	LMS	Mut	0.984	29.5	392			+
IB128	ExOS	WT	0.546	12.036	40	−−−	−	−−
93T449	WDLPS	WT	0.869	19.2	ND	ND	ND	ND

*UPS* undifferentiated pleomorphic sarcoma, *DDLPS* dedifferentiated liposarcomas, *LMS* leiomyosarcomas, *ExOS* extrakeletal osteosarcoma, *WDLPS* well-differentiated liposarcoma

### Reagents

Rucaparib and trabectedin were supplied by Euromedex (Souffelweyersheim, France) and Pharmamar (Madrid, Spain), respectively.

### Cell viability

Antiproliferative and cytotoxic effects of trabectedin and rucaparib were first determined on nine cell lines using Cytation 3 technology (Colmar, France). Briefly, cells were seeded in 384-well plates and were then exposed to trabectedin and/or rucaparib for 72 h. Cells were then marked with propidium iodide (PI) and Syto 24 fluorochromes for 30 min. Quantitative fluorescence and cell imaging were performed with Cytation 3 at *λ* = 617 nm for PI and 521 for Syto 24.

Trabectedin and rucaparib effects on cell viability were also investigated using the MTT assay [3-(4,5-dimethylthiazol-2-yl)-2,5-diphenyl tetrazolium bromide] (Sigma-Aldrich Chimie, Saint-Quentin-Fallavier, France) as an indicator of metabolically active cells. A known number (2000 or 3000) of STS cells was transferred into 96-well plates and incubated for 24 h before the addition of the test compound. The cells were then exposed for 72 h at 37 °C to an increasing concentration range of trabectedin and rucaparib. MTT at a final concentration of 0.5 mg/ml was added, and following incubation for 3 h, formazan crystals were dissolved in DMSO. Absorbance of the colored solution was measured on a microplate-photometer (Bio-Tek Instruments, Colmar, France) using a test wavelength of 570 nm and a reference wavelength of 630 nm. The concentration of substance required for 50% growth inhibition (IC50) was estimated with GraphPad Prism software (GraphPad Software Inc., San Diego, CA, USA).

### Cell cycle analysis

Cell cycle distribution of the four cell lines was studied by examining DNA content using fluorescence-activated cell sorting and analyzed using Cell Quest Pro software (BD Biosciences, San Jose, CA, USA). 2 × 10^5^ cells were seeded in 6-well plates, and after 24 h, the cells were treated for 48 h with two different concentrations of trabectedin and/or rucaparib, centrifuged at 1500 g for 5 min, and washed twice with PBS. The cells were then fixed with 70% ethanol at 4 °C overnight. Following ethanol removal, the cells were washed twice with PBS. Next, 300 μl of a PI and ribonuclease-containing solution were added to the cells and then analyzed by FACS. The data were analyzed with FlowJo v.7.6.3 software, and the results were expressed in terms of percentage of cells in a given phase of cycle.

### Apoptosis

For apoptosis assessment, 1.5 × 10^5^ cells were seeded in 6-well plates. After 24 h, cells were treated with two doses of trabectedin and/or rucaparib for 72 h and exposed to FITC-Annexin V and PI according to the manufacturer’s protocol (BD Biosciences, Erembodegem, Belgium). This allows us to distinguish Annexin V-positive cells in early apoptosis from Annexin V- and PI-positive cells in late apoptosis. Cells were analyzed by flow cytometry using FL1 for Annexin V and FL2 for PI. Flow cytometry (FACScan; BD Biosciences) data were analyzed with FlowJo v.7.6.3 software.

### PARP1 activity

PARP activity was measured in cell extracts using the HT PARP/apoptosis assay (Amsbio, Abingdon, UK) according to the manufacturer’s protocol. Briefly, 5 × 10^3^ cells were seeded in a 96-well plate and exposed to one concentration of trabectedin and/or rucaparib for 48 h. After exposure, protein extracts were prepared, transferred to histone-coated plates, and tested for ribosylation reaction. PARP activity was evaluated by an ELISA method that semi-quantitatively detects poly(ADP-ribose) or PAR. Absorbance was correlated with PARP activity and was measured at 450 nm, and the percentage of inhibition relative to the untreated control was calculated as follows: *C* = net absorbance in the absence of induced apoptosis; *D* = net absorbance determined during apoptosis; % inhibition of PARP = (*C*−*D*)/C*100.

### Confocal microscopy

Cells were seeded on coverslips and treated with one concentration of trabectedin, rucaparib, or a combination of the two drugs for 72 h. The slides were then washed twice with PBS, fixed in 4% formaldehyde, and incubated with anti-phosphoγH2ax monoclonal antibody (Cell Signaling, Leiden, Netherlands) overnight and then with goat anti-rabbit Alexa Fluor 488 antibody (Invitrogen, Paisley, UK). The slides were then counterstained using 4,6-diamidino-2-phenylindole (Hoechst).

### ERCC5, ERCC1, and BRCA1 mRNA expression and genotyping

Total RNA was extracted using an RNeasy kit according to the manufacturer’s instructions. Quantification of gene expression was performed using the ABI Prism 7900HT sequence detection system (Applied Biosystems, Foster City, CA, USA). The following primers and 50 labeled fluorescent reporter dye (6-FAM) probes were used: For β-actin, the forward primer was 5′-TGA GCG CGC CTA CAG CTT-3′, the reverse primer was 5′-TCC TTA ATG TCA CGC ACG ATT T-3′, and the 5′-FAM ACC ACC ACG GCC GAG CGG 3′-tetramethylrhodamine (TAMRA) probe was used. For BRCA1, the forward primer was 5′-GGC TAT CCT CTC AGA GTG ACA TTT TA-3′, the reverse primer was 5′-GCT TTA TCAGGT TAT GTT GCA TGG T-3′, and the minor groove binder (MGB) 5′-FAM CCA CTC AGC AGA GGG-3′ nonfluorescent quencher (NFQ) probe was used. For ERCC1, the forward primer was 5′-GGG AAT TTG GCG ACG TAA TTC-3′, the reverse primer was 5′-GCG GAG GCT GAG GAA CAG-3′, and the 5′-FAM CAC AGG TGC TCT GGC CCA GCA CAT A 3′-TAMRA probe was used. For ERCC5, the forward primer was 5'-GAA GCG CTG GAA GGG AAG AT-3′, the reverse primer was 5′-GAC TCC TTT AAG TGC TTG GTT TAA CC-3′, and the MGB probe 5′-FAM CTG GCT GTT GAT ATT AGC ATT 3′-NFQ was used. Relative gene expression was calculated according to the comparative ΔΔCt method using β-actin as an endogenous control and commercial RNA controls (Stratagene, La Jolla, CA; Applied Biosystems) as calibrators.

### Genomic index calculation

The genomic index (GI) was calculated for each profile of cell lines as follows: GI = *A*_2_/*C*, where *A* is the total number of alterations and *C* is the number of involved chromosomes.

### In vivo study

#### Cell lines xenografts

Four- to five-week-old female Ragγ2C-/- mice were used. Induction of tumor xenografts was performed by subcutaneous injection of 0.2 ml cell suspensions containing 5 × 10^6^ live IB115 cells or by subcutaneous implantation of UPS tumor fragment (PDX) into the right flank of the mice. This study followed the Spanish and European Union guidelines for animal experimentation (RD 1201/05, RD 53/2013, and 86/609/CEE, respectively). Mice were randomized into control and treatment groups (*n* = 8 for vehicle and rucaparib groups and *n* = 12 for trabectedin and combination groups for IB115 and *n* = 5 for vehicle and rucaparib groups and *n* = 8 for trabectedin and combination groups in PDX) 2 weeks after the tumor became measurable (15 days after injection: day 1 of treatment). Mice were randomized in four groups: vehicle (NaCl0.9%), trabectedin alone (0.05mk/kg IV once a week), rucaparib alone (10 mg/kg IP five times per week), and both drugs (trabectedin once a week and rucaparib five times per week at 0.05 mg/kg and 10 mg/kg, respectively). Trabectedin and rucaparib were administered using 0.9% NaCl as the vehicle. The tumors were measured every 2–3 days with a caliper, and diameters were recorded. Tumor volumes were calculated using the formula: *a* ^2^ *b*/2, where a and b are the two largest diameters. The mice were sacrificed by cervical dislocation 1 week after treatment arrest, and the tumors were collected for histopathological analyses. Progression-free survival curves were established based on twofold tumor increase as event. All experimental manipulations with mice were performed under sterile conditions in a laminar flow hood. After the sacrifice of the mice, tumors were harvested in 10% paraformaldehyde. Tissue pictures was carried out with an Olympus CKX41 (×2.5) using image capture cellSens Entry software version 1.14 (Olympus, Rungis, France) for Windows, and percentage of necrosis was estimated by an anatomical pathologist.

### Statistical analysis

Data were analyzed using the Student *t* test for comparison of two means and ANOVA followed by the Turkey’s multiple comparison tests for more than two groups; all the experiments were repeated in duplicate or triplicate. Data are represented as mean ± SD, and significant differences are indicated as *p < 0.05, **p < 0.01, and ***p < 0.001.

The analysis of progression-free survival was using LogRank test (Mantel-Cox test).

## Results

### Antiproliferative activity of trabectedin and rucaparib in STS cell lines

We studied the sensitivity of nine STS cell lines to trabectedin and rucaparib. The IC50 values for trabectedin (Et-743) and rucaparib are shown in Table 1. All of the cell lines were highly sensitive to trabectedin, with IC50 values ranging between 0.352 and 2.64 nM. Three out of nine cell lines were sensitive to rucaparib, with IC50 values ranging between 1.104 and 1.488 μM. The other cell lines were relatively resistant to rucaparib, with IC50 values ranging between 12 and 58.08 μM. We did not find any correlation between the expression status of DNA repair genes (*ERCC1*, *ERCC5*, *BRCA1*), genomic index, or mutational status of *TP53* (wild-type or mutated) of the STS cells and sensitivity to rucaparib (Table 1).

### Rucaparib blocks basal and trabectedin-induced PARP-1 enzymatic activity in leiomyosarcoma cells

**Fig. 1:**
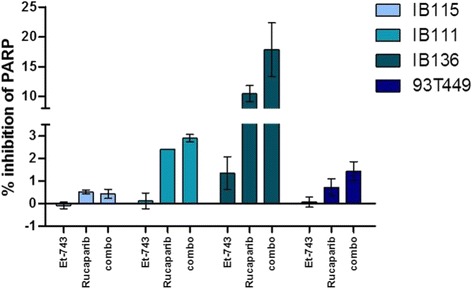
PARP activity measured during apoptosis. Percentage of PARP activity inhibition in IB111, IB115, IB136, and 93T449 after 48 h of treatment with 0.001, 0.00015, 0.007, or 0.00005 μM of trabectedin, respectively; 10, 1.3, 13, or 1 μM of rucaparib, respectively; or both drugs in combination

PARylation significantly triggers the accumulation of several DNA damage response (DDR) proteins at DNA lesions and is, therefore, a marker of DNA damage. We evaluated the effects of trabectedin, rucaparib, and combination in PAR synthesis after 72 h of incubation to determine the extent of this effect. As expected, the rucaparib inhibited basal PARP-1 activity (reducing the amount of PARylated proteins) in all cell lines, and we observed an effect of trabectedin and combination of drugs only in leiomyosarcoma cells (IB136) (Fig. 1).

### Trabectedin and rucaparib combination increases DNA damage

**Fig. 2:**
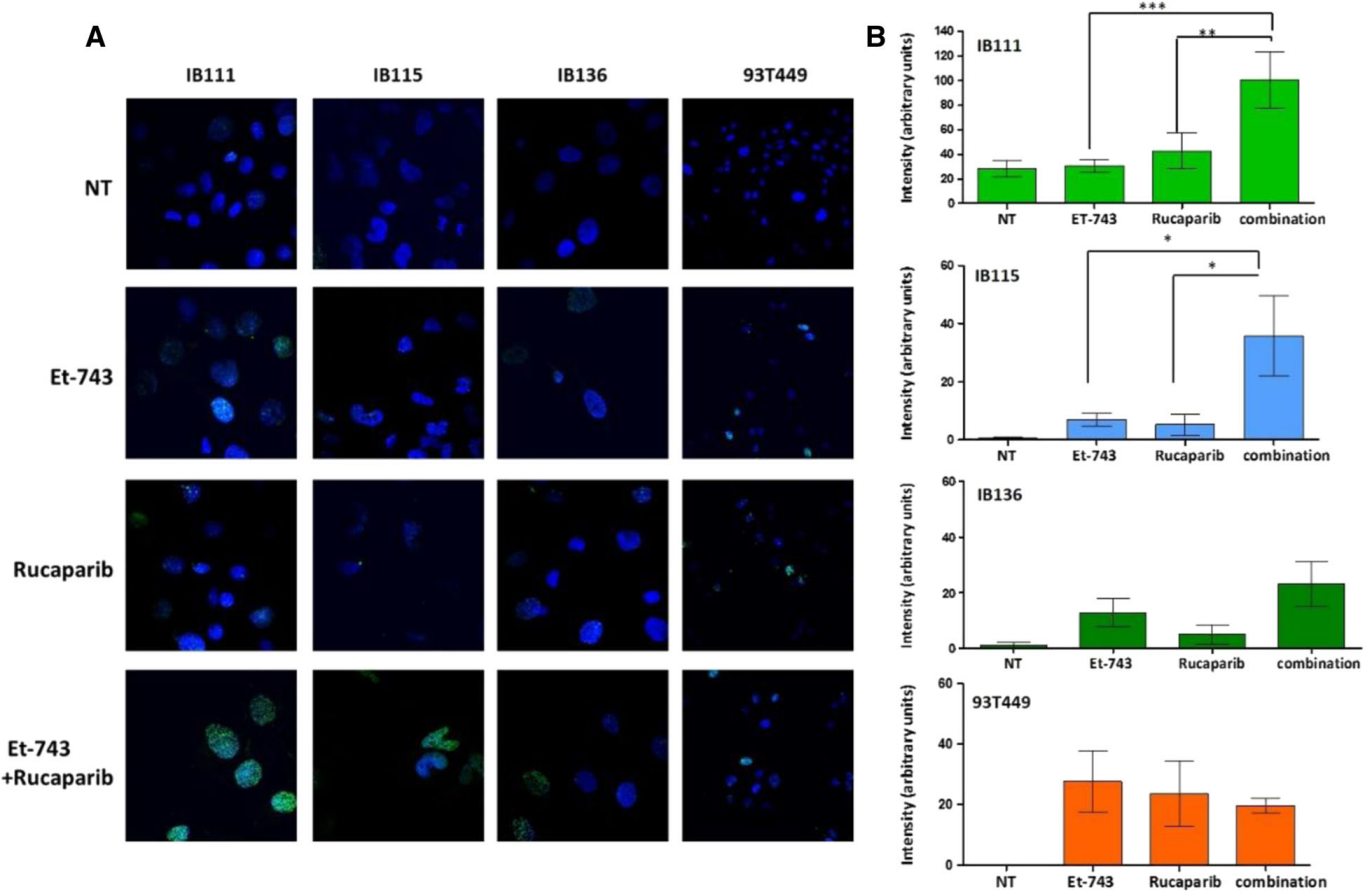
**a** IB111, B115, IB136, and 93T449 cells were immunostained with anti-P-γH2AX-specific antibodies before and after treatment with trabectedin at 0.001, 0.000075, 0.0035, and 0.00005 μM, respectively; rucaparib at 10, 1.3, 13, and 1 μM, respectively; or both drugs in combination. **b** Quantification of P-H2AX punctae in IB115, IB111, IB136, and 93T449 cell lines

To quantify the extent of DNA damage, we also analyzed γ-H2AX expression after the different drug treatments using confocal microscopy. As shown in Fig. 2, the combination of trabectedin (Et-743) and rucaparib induced significantly higher levels of γ-H2AX expression only in two cell lines IB115 and IB111 cell lines. The expression of γ-H2AX was evident even with various concentrations of trabectedin as a single agent, which prevented the formation of DSBs.

### Trabectedin and rucaparib are synergistic in STS cell lines

We studied the effects of the combination of rucaparib and trabectedin. Nine STS cell lines were exposed during 72 h to different combinations of both agents at a constant ratio of 1: trabectedin and rucaparib were mixed and diluted serially (usually twofold serial dilutions with several concentrations above and below the IC50 for the two drugs), and combination indices (CIs) were determined according to Chou et al. [24]. The results are described in Table 2. Interestingly, we observed an additive or synergistic effect when using the MTT method in 80% of the STS cell lines (in particular in liposarcomas).

**Table 2:** Trabectedin plus rucaparib combination study: combination index according to Chou and Talalay

Cell lines	Combination index	Comments
IB114	0.64	Synergistic
IB115	0.75	Synergistic
IB111	0.71	Synergistic
IB133	1.17	Antagonist
IB134	1.02	Additive
IB136	1.18	Antagonist
IB112	0.99	Additive
IB128	0.92	Additive
93T449	0.86	Synergistic

### Trabectedin and rucaparib combination induces apoptosis and cell cycle arrest in STS cell lines

**Fig. 3:**
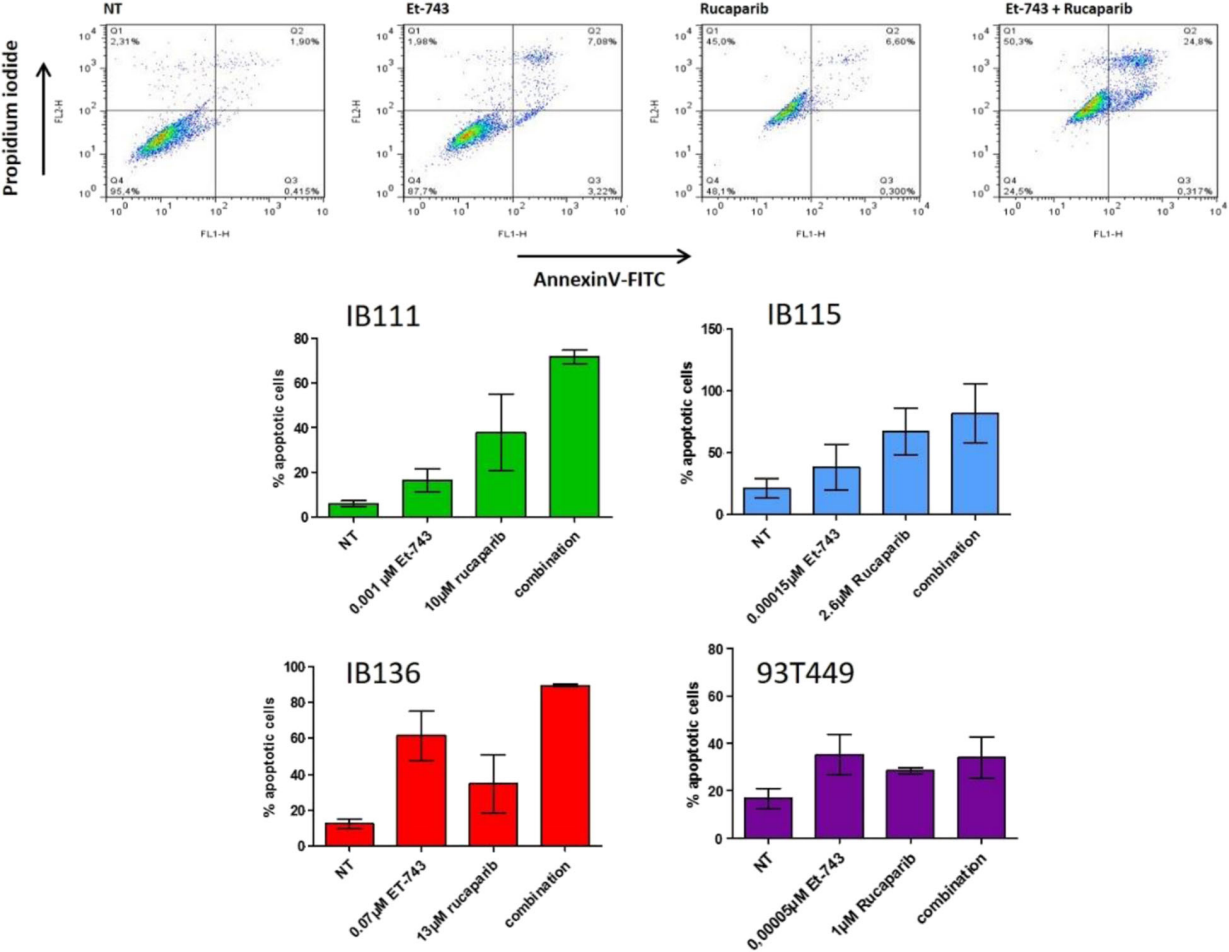
Effect of trabectedin (Et-743) and rucaparib combination on apoptosis **a** Annexin V FITC-A vs propidium iodide-A plots from the gated cells shows the populations corresponding to viable and non-apoptotic (Annexin V–PI–), early (Annexin V + PI–), and late (Annexin V + PI+) apoptotic cells in IB111 cell line. **b** Quantification of apoptotic cells after 72 h of treatment with trabectedin or rucaparib alone or combination of the two drugs

**Fig. 4:**
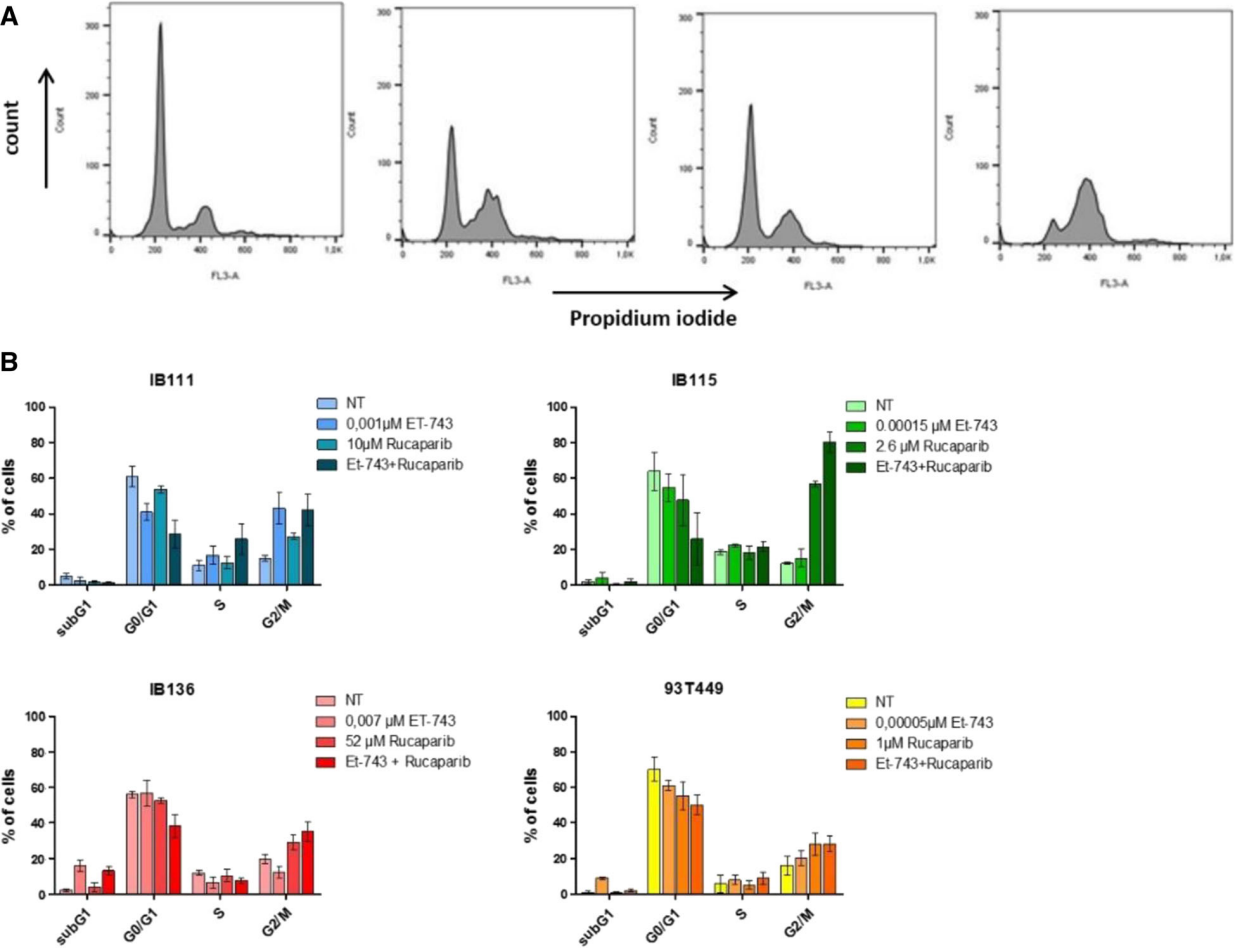
Effect of trabectedin (Et-743) and rucaparib combination on cell cycle progression in four STS cell lines: IB111, IB115, IB136, and 93T449. **a** Cell-cycle profile after 48 h of treatment with trabectedin and/or rucaparib analyzed by PI incorporation and flow cytometry in the IB111 cell line. **b** Cell-cycle distribution was calculated from the flow cytogram.

We studied the effects of trabectedin and rucaparib combination on apoptosis induction after 72 h of drug exposure as well as cell cycle effects after 48 h of treatment in the cell lines IB115, IB111, IB136, and 93T449. We observed that the drug combination (picomolar amounts of trabectedin and micromolar amounts of rucaparib) increased the rate of apoptosis in comparison with the drugs alone in IB111 and IB136 cell lines (Fig. 3). Furthermore, G2/M accumulation and a decrease in the G0/G1 peak were also observed after treatment with the drug combination (Fig. 4), particularly in the IB115 cell line.

### Trabectedin and rucaparib combination reduces tumor growth in vivo

**Fig. 5:**
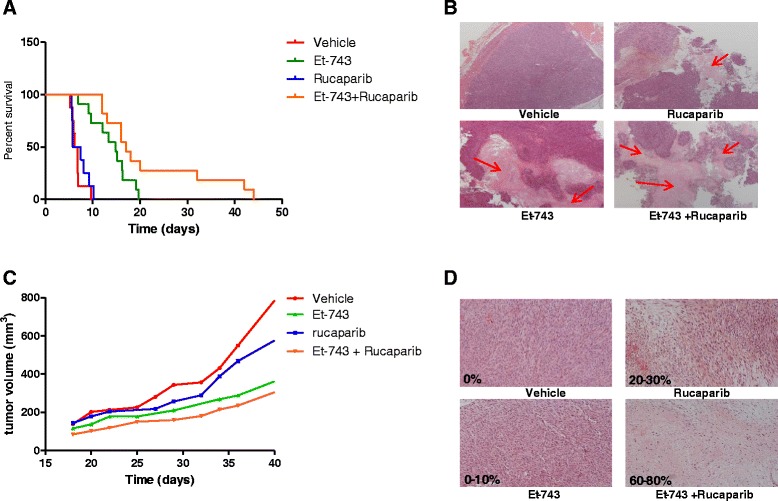
**a** In vivo effect of trabectedin (Et-743) and rucaparib combination. The doubling time was calculated from the tumor progression curve. **b** Tissue pictures of four representative tumors, the *red arrows* show the necrotic areas. **c** Effect of combination of trabectedin (Et-743) and rucaparib on a PDX model of UPS. **d** tissue pictures of four representative tumors

To further validate in vitro study, we performed in vivo studies to test the antitumor effects of the trabectedin and rucaparib combination. Xenografts were generated by subcutaneous injection of IB115 cells in ragγ2C-/- mice or by subcutaneous implantation of UPS tumor fragment (PDX). Animals were randomized in four groups and treated for 3 weeks. These groups included control (NaCl 0.9%), trabectedin (trabectedin alone; 0.05 mg/kg IV once a week), rucaparib (rucaparib alone; 10 mg/kg BID IP, five times per week), and combination. After 3 weeks of treatment, we observed a significant effect on progression-free survival (evaluated as the time span from the treatment start and the doubling of the initial tumor volume); median time to doubling was 17.1 days for combination, 14.8 days for trabectedin (*p* = 0.045), and 6.6 days for rucaparib (*p* < 0.0001) (Fig. 5b) in IB115 xenografts model. After 3 weeks of treatment, the mice were sacrificed and tumors were extracted, weighed, and evaluated by histopathology. No signs of toxicity were observed with the combination treatment. Evaluation of percentage of necrosis indicates a good relationship between necrosis and treatment efficacy; for the combination, there are 25% of tumors with at least 60% of necrosis while only 0 or 10% for vehicle and drugs alone. We observed the same results in UPS PDX model; the combination regimen reduced tumor volume in comparison with single agent (Fig. 5c) and evaluation of necrosis indicate, as well as in IB 115 xenografts model, a good correlation with treatment (Fig. 5c).

## Discussion

Trabectedin has been recently approved in the USA and Europe for the management of advanced STS in patients who have failed to benefit from anthracycline-containing regimens. However, the activity of this drug as a single agent is limited, with a median PFS of only 4 months. Thus, there is a need for a more active regimen for use in STS patients.

Several studies suggest that PARP inhibition may be relevant to treating soft-tissue sarcomas. For instance, it is well known that loss of BRCA-1 or BRCA-2 leads to sensitivity to PARP1 inhibition, resulting in apoptosis. Xing et al. reported that 29% of uterine leiomyosarcomas had decreased or completely absent BRCA-1 protein expression, which is postulated to be due to methylation of the BRCA-1 gene promoter [25]. Schoffski et al. reported a decrease in BRCA-1 expression in 50% of soft-tissue sarcoma samples [26].

In addition, members of the Fanconi family of proteins are involved in double-strand DNA repair through activation of ATM and ATR and formation of a nuclear complex of five Fanconi family proteins. This complex subsequently co-localizes with BRCA1 and BRCA2 for DNA repair [27]. Loss of function or expression of any of these proteins or “BRCA-ness” confers sensitivity to PARP1 inhibition [28, 29]. ATM loss has been reported in several sarcoma subtypes, such as leiomyosarcoma and rhabdomyosarcoma [30, 31]. Finally, loss of PTEN confers sensitivity to PARP1 inhibition [32]. This molecular aberration is a crucial event in tumorigenesis of leiomyosarcoma [33] and occurs frequently in dedifferentiated liposarcomas [34], the most frequent sarcoma subtype.

Several pre-clinical studies have shown that combining PARP inhibitors with methylating agents (DTIC, temozolamide), alkylating agents (cyclophosphamide, ifosfamide), or doxorubicin may help treat soft-tissue sarcomas by increasing antitumor efficacy [35–38]. We have also reported that BRCA1 genotype status was predictive of trabectedin efficacy in patients with advanced STS [19, 20]. For all these reasons, we decided to investigate whether the combination of PARP inhibition with trabectedin confers additive or synergistic antitumor activity.

Our results show that the combination of trabectedin and rucaparib was synergistic, increasing apoptotic activity and arresting cell cycle at the G2/M phases in STS, in particular dedifferentiated liposarcomas, while we did not observed a synergistic effect in leiomyosarcomas. One possible explanation is that our LMS cell lines were P53 mutated, and it has been shown that trabectedin proapoptotic activity involve mainly P53 [39].Furthermore, we demonstrated that although both agents alone induced DNA damage through an accumulation of γH2AX foci in vitro, the combined use or trabectedin and rucaparib significantly increases this effect. We also observed this synergistic antitumor activity in vivo, where the drug combination increased significantly progression-free survival in comparison with trabectedin and rucaparib used as single agents.

## Conclusion

In conclusion, to the best of our knowledge, we report here the first pre-clinical evidence that the combination of a PARPinh and trabectedin is synergistic in soft-tissue sarcomas. Interestingly, promising activity of this combination has also been observed in bone sarcomas [40]. Our results are sufficient to design a clinical study with the aim of assessing the combination of PARPinh and trabectedin in the treatment of STS.
